# Desiccation and Mortality Dynamics in Seedlings of Different European Beech (*Fagus sylvatica* L.) Populations under Extreme Drought Conditions

**DOI:** 10.3389/fpls.2016.00751

**Published:** 2016-06-14

**Authors:** Andreas Bolte, Tomasz Czajkowski, Claudia Cocozza, Roberto Tognetti, Marina de Miguel, Eva Pšidová, Ĺubica Ditmarová, Lucian Dinca, Sylvain Delzon, Hervè Cochard, Anders Ræbild, Martin de Luis, Branislav Cvjetkovic, Caroline Heiri, Jürgen Müller

**Affiliations:** ^1^Thünen Institute of Forest EcosystemsEberswalde, Germany; ^2^Instituto per la Protezione Sostenibile delle Piante (IPSP), Consiglio Nazionale delle RicercheSesto Fiorentino, Italy; ^3^Dipartimento di Bioscienze e Territorio, Università del MolisePesche, Italy; ^4^EFI Project Centre on Mountain Forests (MOUNTFOR), Edmund Mach FoundationSan Michele all'Adige, Italy; ^5^BIOGECO, INRA, Université de BordeauxCestas, France; ^6^Institute of Forest Ecology, Slovak Academy of ScienceZvolen, Slovakia; ^7^Marin Dracea National Forest Research-Development InstituteBucharest, Romania; ^8^PIAF, INRA, Université Clermont AuvergneClermont-Ferrand, France; ^9^Department of Geosciences and Natural Resource Management, University of CopenhagenFrederiksberg C, Denmark; ^10^Grupo de Clima, Agua, Cambio Global y Sistemas Naturales, Departamento de Geografía y Ordenación del Territorio, Facultad de Filosofía y Letras, Instituto de Investigación en Ciencias Ambientales, Universidad de ZaragozaZaragoza, Spain; ^11^Faculty of Forestry, University of Banja LukaBanja Luka, Bosnia and Herzegovina; ^12^Swiss Federal Institute for Forest, Snow and Landscape Research WSLBirmensdorf, Switzerland

**Keywords:** *Fagus sylvatica*, drought, desiccation, mortality, LD50_SWA_, soil water availability, genetic variation, pre-dawn water potential

## Abstract

European beech (*Fagus sylvatica* L., hereafter beech), one of the major native tree species in Europe, is known to be drought sensitive. Thus, the identification of critical thresholds of drought impact intensity and duration are of high interest for assessing the adaptive potential of European beech to climate change in its native range. In a common garden experiment with one-year-old seedlings originating from central and marginal origins in six European countries (Denmark, Germany, France, Romania, Bosnia-Herzegovina, and Spain), we applied extreme drought stress and observed desiccation and mortality processes among the different populations and related them to plant water status (predawn water potential, Ψ_PD_) and soil hydraulic traits. For the lethal drought assessment, we used a critical threshold of soil water availability that is reached when 50% mortality in seedling populations occurs (LD50_SWA_). We found significant population differences in LD50_SWA_ (10.5–17.8%), and mortality dynamics that suggest a genetic difference in drought resistance between populations. The LD50_SWA_ values correlate significantly with the mean growing season precipitation at population origins, but not with the geographic margins of beech range. Thus, beech range marginality may be more due to climatic conditions than to geographic range. The outcome of this study suggests the genetic variation has a major influence on the varying adaptive potential of the investigated populations.

## Introduction

There is much evidence that ongoing climate change is warming the global climate system given the average temperature rise of 0.85°C for the combined land and ocean surface over the period from 1880 to 2012. And there is strong evidence that an increased frequency of extreme weather events like heat waves and precipitation extremes is linked to global warming (Coumou and Rahmstorf, 2012). Projections of further warming in the Twenty-first century are linked to a likely increase in, and intensification of, heat waves and drought periods, in particular toward the end of the century (IPCC, 2013). For Europe it has been found, that the severity, duration, and frequency of drought events increased from 1950 to 2012 in Mediterranean regions, but moderately also in parts of Central Europe (Spinoni et al., 2015). Similar tendencies are also projected for the future (IPCC, 2012; Stagge et al., 2015). European forests have already responded to more intensive drought impacts with increased mortality (Allen et al., 2010).

The natural vegetation in Central Europe and higher elevated areas in southern Europe is dominated largely by European beech (*Fagus sylvatica* L.; Bohn et al., 2004; Bréda et al., 2006). Besides being of high economic value, beech is also of ecological importance, since it is the dominant tree species in many forest ecosystems (Leuschner et al., 2006). Beech can grow well on a wide variety of sites except on extremely dry soils with low water storage capacity, stagnic soils, or soils prone to flooding and high ground water table (Ellenberg and Leuschner, 2010). Beech is dominant in many deciduous forests in Europe under maritime and temperate climate conditions with mild winters and moist summer conditions; the pronounced cold, dry, and continental climate limits its distribution (Bolte et al., 2007). As a distinct shade-tolerant tree species, beech itself reduces below-canopy irradiance often below 5% of the open field irradiance (Emborg, 1998; Collet et al., 2001), giving it the competitive advantage over other tree species (Ellenberg and Leuschner, 2010).

However, beech is generally reputed to be sensitive to drought (e.g., Aranda et al., 2000; Gessler et al., 2007) and could lose its competitive advantage to less drought-sensitive species like sessile oak [*Quercus petraea* (Matt.) Liebl.] under water-limited conditions (Scharnweber et al., 2011). Looking on direct drought impact, beech's vulnerability to cavitation seems to make it extremely sensitive to singular extreme water deficit, and hence to drought (Barigah et al., 2013; Urli et al., 2013). A critical internal water status in beech seedlings is reached at shoot water potential of –1.9 MPa (Hacke and Sauter, 1995) upon which a loss of hydraulic conductivity may eventuate. In case of continuing drought, 50% loss of hydraulic conductivity (P50) can occur between –2.0 MPa and –3.0 MPa (Cochard et al., 1999; Cruiziat et al., 2002). A critical loss of hydraulic conductivity (P88) was found at –4.2 MPa (Urli et al., 2013), and Barigah et al. (2013) reported 50% mortality among beech seedlings at –4.5 MPa plant water potential (xylem pressure). During the extreme drought year 2003, Granier et al. (2007) identified 40 and 20% of relative available soil water content as thresholds below which gross primary production, and total ecosystem respiration decreased respectively. However, there is still no coherent approach to link quantitatively the environmental drought impact, e.g., assessed as soil water deficit, to the desiccation and mortality of tree seedlings. Bréda et al. (1995) and Czajkowski et al. (2009) demonstrated that plant water status can be linked to soil matrix potential at the lower end of the effective rooting zone (ERD). Accordingly, a simultaneous study of soil hydraulic traits and desiccation dynamics may link plant mortality to soil water deficit, not at an individual, but also at a mean population level. Such an indicator can be applied in regional assessments and projections on soil water availability and critical drought risk (e.g., Bolte, 2015).

In Central Europe, beech exhibits high genetic diversity within populations (Vornam et al., 2004), but genetic differentiation between populations is also evident at continental scale (Magri et al., 2006; Dounavi et al., 2016). Accordingly, several studies on young beech seedlings revealed remarkable differences in the adaptive potential of different beech populations to drought: beech populations from the xeric sites and/or range margins seem to have a higher drought tolerance than those from mesic sites and/or central ranges (Italy: Tognetti et al., 1995; Bosnia and Herzegovina: Ivojević et al., 2012; Slovakia: Pšidová et al., 2015; Germany: Schraml and Rennenberg, 2002; Peuke et al., 2002; Poland and Germany: Czajkowski and Bolte, 2006a; Rose et al., 2009; Spain, Bulgaria and Germany: Thiel et al., 2014; Germany, Balkan peninsula, Bulgaria and Greece: Dounavi et al., 2016). This response could be due to population dynamic processes at the southern and eastern margins of the beech distribution range including local, evolutionary adaptation to increasing drought stress on xeric sites (Hampe and Petit, 2005).

Except for the regional study by Ivojević et al. (2012), the previous experimental studies on population level focused on seedling growth performance, hydraulic traits, and/or water status under moderate or severe drought, but did not systematically apply severe drought, which induces mortality among the seedlings. Thus, a continental study of beech seedling mortality induced by extreme drought events and variation in mortality among populations level is lacking. Using the pan-European EU Cost STReESS network, we collected seeds from seven sites in six European countries throughout the native beech range and conducted a common garden experiment in Germany to (1) derive a desiccation and mortality indicator at the population level that can be related to soil water availability (SWA), (2) derive critical limits of soil water availability (SWA) for the studied beech populations, and (3) reveal possible population variation in extreme drought response and desiccation.

## Materials and methods

### Plant material

**Figure 1:**
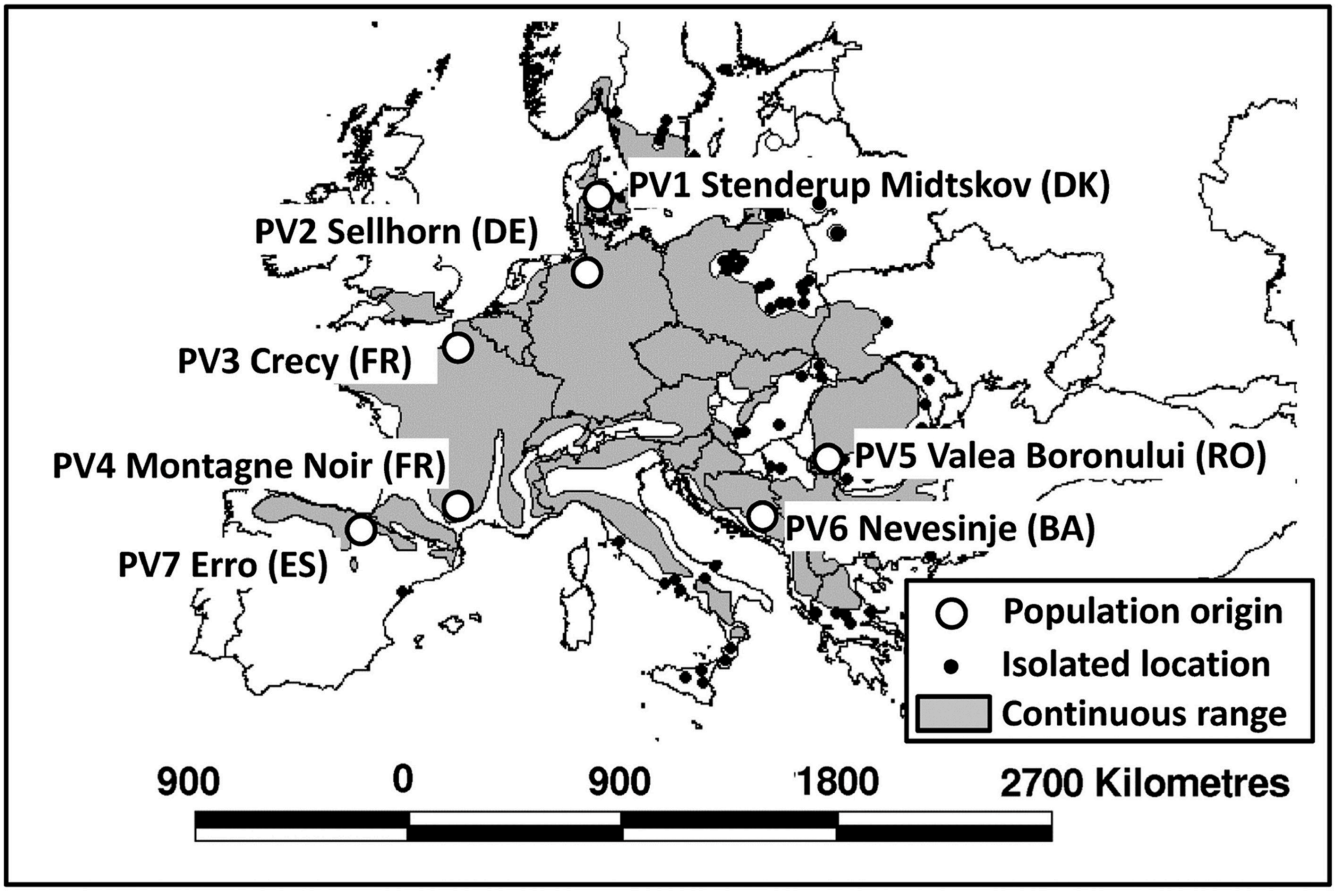
**Location of the origins of the investigated populations (circles), and continuous distribution range of European beech (gray area) based on the distribution map of Bolte et al. (2007)**.

For the experiments, we collected at least 1 kg of fresh beech seeds from four different autochthonous, old-growth beech stands [location see Table 1; population Stenderup Midskov (Denmark), Nevesinje (Bosnia), Valea Boronului (Romania), and Erro (Spain)]. The collected seeds originated from at least ten different old-growth beech individuals. Seeds from three other populations originated from commercial seedbanks (Crecy and Montagne Noir, France) and Sellhorn (Germany), which were also collected in single stands. The stand locations cover a large variety of environments within the natural beech distribution range, in particular including geographically marginal sites (Figure 1). For the climatic characterization of the population origins, we used temperature and precipitation parameters and the Ellenberg Climate Quotient *EQ* (Ellenberg, 1988, Equation 1):

(1) $$\begin{array}{l} EQ=\left(\frac{T_{max.}}{P_{year}}\right)\cdot 1000 \end{array}$$

where *T*_*max*__._ is the mean temperature in the warmest month (°C) and *P*_*year*_ the total annual precipitation (mm).

**Table 1:** **Temperature (T) and precipitation (P) [year, growing season from April (4) to September (9)] at the origin of the seedling populations, derived from WorldClim grid data (Source: http://www.worldclim.org/current, period 1950–2000, ESRI grid, resolution 30 s, ca. 1 km^2^)**.

**No**.	**Population**	**Country**	**Lat. N (°)**	**Long. E (°)**	**Alt. asl. (m)**	**T year (°C)**	**T 4–9 (°C)**	**Tmax. (°C)**	**P year (mm)**	**P 4–9 (mm)**	**EQ^*a*^**	**Am^*b*^**
PV1	Stenderup Midtskov	DK	55.47	9.65	18	7.7	11.2	15.8	720	352	21.9	40.7
PV2	Sellhorn	DE	53.35	9.93	86	8.2	12.1	16.9	748	402	22.6	41.1
PV3	Crecy	FR	50.25	1.88	30	10.5	13.7	17.5	637	291	27.3	31.1
PV4	Montagne Noir	FR	43.50	2.22	341	12.4	16.1	20.7	791	376	26.2	35.3
PV5	Valea Baronului	RO	44.77	21.68	445	9.3	14.5	19.6	722	424	27.0	37.4
PV6	Nevesinje	BA	43.27	18.13	862	9.6	13.7	18.8	1199	493	15.7	61.2
PV7	Erro	ES	43.00	-1.47	931	9.1	12.9	17.2	1166	511	14.7	61.0

*Altitude values represent the means of the 30 s grid cell*.

a *EQ: Ellenberg Climate Quotient*.

b *Am: Aridity index of De Martonne*.

Moreover, we applied De Martonne (1926) Aridity index *Am* (Equation 2):

(2) $$\begin{array}{l} Am=\left(\frac{P_{year}}{T_{year}+10}\right) \end{array}$$

with the annual mean temperature *T*_*year*_ (°C). The found ranges of climatic parameters (Table 1) cover quite well the climatic range limits of European beech reported by Fang and Lechovicz (2006) with e.g., *T*_*year*_ (°C) ranging from 7.2 to 13.5 and *EQ* from 16.8 to 29.0, but not reaching the absolute xeric extremes. However, our exceedance of *EQ* limits on higher elevated sites (PV6, PV7) may indicate the problem to adequately characterize both lowland and mountainous climatic limits with indices mainly based on annual means, only.

The seeds were collected in autumn 2013, stored and transported in cool, dry environments to the Thünen Institute of Forest Genetics in Groß-Hansdorf (Germany). Uniformly sized seeds of each population were surface-sterilized by soaking in 3% sodium hypochlorite for 5 min and rinsing with deionized water. Thereafter, a stratification procedure was performed: (1) the seed moisture content was reduced to about 8% of the fresh seeds' moisture content (e.g., by storing them ≈1 week in a cool, dry place), (2) seeds were preserved in plastic bags in a freezer at −5°C until mid-February (stratification by frost), (3) the seed moisture was increased at a temperature of 3–5°C (using a water sprayer); (4) as soon as the first little sprout was visible, the seedlings were transplanted into pots. With this procedure at least 200 individual seedlings per population were available for the drought experiments.

After the success of seed germination was recorded, plants were cultivated in cylindrical PVC pots (1.4 liters) filled with 70% silty sand (grain size 0–2 mm), 30% peat-based substrate mixed with with 2 kg m^−3^ Osmocote (NPK 14:13:13+7SO_3_, plus micro elements). Plants grew under slightly reduced open field light conditions (≈ 70% rel. open field irradiance) in a greenhouse environment.

After transferring the seedlings to the Thünen Institute of Forest Ecosystems in summer 2014, a drought simulation was carried out in late summer 2014 in a greenhouse at the University of Sustainable Development (HNE) in Eberswalde (52°49′28 ” N 13°47′29” E, 30 m a.s.l.). Within the treatment period relative air humidity averaged 69%, with a minimum of 30% and a maximum of 88%. Air temperature ranged between 11°C (minimum during night) and 31°C (maximum during day), and attained a mean of 19.0°C. The plants grew under ambient light conditions during the experiment without any additional illumination. During the experiment the light intensity never exceeded 1000 μmol photons m^−2^ s^−1^ under sunny conditions.

Plant traits before the commencement of the drought experiment (Table 2) show some variation in root collar diameter, plant height and leaf number among the populations, but a common pattern across populations was not observed. No significant differences were found for total leaf area.

**Table 2:** **Means (± standard error) of plant traits for the beech seedlings before the drought stress experiment**.

**No**.	**Population**	**Country**	**Root collar diameter (mm)**	**Plant height (cm)**	**Leaf area (cm^2^)**	**Leaf number (n)**
PV1	Stenderup Midtskov	DK	2.00^b^ ± 0.00	14.67^b^ ± 0.32	77.11 ± 5.47	9.50^b^ ± 0.90
PV2	Sellhorn	DE	2.44^a^ ± 0.09	12.44^b^ ± 0.32	99.62 ± 12.70	13.13^a,b^ ± 2.38	
PV3	Crecy	FR	2.25^a^ ± 0.10	17.50^a^ ± 0.40	99.44 ± 12.19	12.75^a,b^ ± 1.50	
PV4	Montagne Noir	FR	2.45^a^ ± 0.11	14.92^a, b^ ± 0.76	69.99 ± 11.50	4.40^c^ ± 0.57
PV5	Valea Baronului	RO	2.00^b^ ± 0.00	15.42^a^ ± 0.47	111.25 ± 16.12	15.00^a^ ± 1.64
PV6	Nevesinje	BA	2.75^a^ ± 0.10	16.77^a^ ± 0.79	110.43 ± 7.47	15.63^a^ ± 1.64
PV7	Erro	ES	2.00^b^ ± 0.00	13.80^b^ ± 0.45	90.12 ± 11.61	9.63^b^ ± 2.27

*Means followed by different letters are significantly different at p < 0.05 (ANOVA, test of population differences, comparison downwards), means of leaf area are not significantly different*.

### Experimental set-up

For the experimental drought simulation, 100 seedlings per population were arranged in the two variants: “control” (C) in 20 pots and “drought treatment” (D) in 80 pots. The seedlings of the different populations were kept together in groups on trolleys in the greenhouse, but population groups were randomly moved and thus spatially re-arranged every 3 days. The group of “control” seedlings was maintained close to field capacity (FC) by frequent watering; whereas, water supply was suspended for those seedlings subjected to drought treatments. Before beginning the drought experiment, initial soil water content and soil dry weight was assessed by weighing samples of the used soil substrate before and after oven drying at 105°C for 48 h. Pots then were watered to saturation. After excess water had drained away, field capacity (FC, Blume et al., 2016) was reached at around –0.06 MPa soil water potential (pF 1.8), and the initial field capacity (FC) pot weight was derived. By subtracting the soil dry weight from FC pot weight we derived the initial soil water content at field capacity. Subsequent changes in pot weight were attributed to changes in soil water content.

The available water capacity (θ_AWC_) of the soil was derived using following Equation (3, cf. Veihmeyer and Hendrickson, 1927).

(3) $$\begin{array}{l} \theta_{\text{AWC}}\;=\;\theta_{\text{FC}}\;-\;\theta_{\text{PWP}}, \end{array}$$

where θ is the soil water content [g] at field capacity (FC, pF 1.8 ≈ −0.06 MPa soil water potential) and at the permanent wilting point (PWP, pF 4.2 ≈ −1,5 MPa soil water potential). θ_PWP_ was derived from a soil water characteristic (pF) curve established for the used soil substrate. With this definition we follow the concept of Reid et al. (1984) who induced the term available soil water for laboratory assessments in contrast to extractable soil water for field estimates (Ritchie, 1981).

The residual soil water availability (SWA) [%] (Equation 4) is defined as the actual soil water content (θ_t_) [g] during drought treatment expressed as a percentage of the initial available soil water capacity (θ_AWC_) [g], and corresponds to the relative extractable soil water (REW) in field studies, Granier et al., 2007):

(4) $$\begin{array}{l} SWA=\frac{\theta_{t}}{\theta_{AWC}} \end{array}$$

To assess SWA, each pot was weighed three times per week after watering was stopped. The treatment started in mid-summer (06/08/2014) and lasted for 8 weeks until all seedlings were considerably desiccated.

### Desiccation and mortality assessments

During the drought treatment, the advanced plant desiccation process was monitored by measuring individual pre-dawn leaf water potentials (Ψ_PD_) with the Scholander chamber technique (Scholander et al., 1964, using the Plant Moisture Vessel *Skye SKPM 1400, Skye Instruments, Llandrindod Wells, UK*). Ψ_PD_ was measured between 0:00 and 5:00 (UT). Seedlings with first optical signs of wilting were measured during the desiccation process. They were regarded as dead when signs of complete wilting occurred with yellow-brown discoloration of the entire leaf surface. To control the status of complete cavitation (>88% loss of hydraulic conductivity at MPa < –6 MPa) we measured pre-dawn water potential of the wilted plants.

The completely wilted plants were separated from the treatment group and re-watered. The date of obvious mortality was recorded. This mortality definition neglects the possibility of wilted beech seedling resprouting after re-watering that were assessed in the following spring 2015. However, the majority of the few found resprouted beech plants died in the days and weeks later due to unspecific reasons which made the viability re-assessment unreliable.

### Derivation of critical drought LD50

For comparing the mortality dynamics of the different beech seedling populations, we adopted the approach for drought impact analyses by Kursar et al. (2009). Due to this, the median lethal desiccation (LD_50_) describes the drought impact that leads to 50% mortality in the seedling population in comparison to the control treatment (cf. also Ivojević et al., 2012). In our study, LD50_SWA_ defines the drought impact as the residual soil water availability (SWA [%]), which is linked to a 50% mortality rate in the population according to previously reported mortality definition.

The critical soil water availability (LD50_SWA_) per seedling population (drought treatment) was derived from a dose-response analysis of mortality rate *M* (Equation 5) and survival rate *S* (Equation 3) as a function of soil water availability (*SWA*) depletion over time:

(5) $$\begin{array}{l} M_{SWA}=\frac{\sum_{{SWA}_{i}}^{SWA}m_{a}}{n_{a}}, \end{array}$$

where *m_a_* is the number of dead plants *m* in population *a*, *n_a_* is the number of total plants per population *a* in the drought experiment (*n_a_*) and period between initial soil water availability *SWA_i_* and current soil water availability *SWA*.

The survival rate *S* (Equation 6) was then calculated from the mortality rate *M*:

(6) $$\begin{array}{l} S_{SWA}=1-M_{SWA} \end{array}$$

The survival rate *S* (range 0–1) was fitted by a non-linear regression analysis applying the software package SAS JMP 11.0 (SAS Institute Inc, 2014). For this we used a two-parameter logistic model (2PL) of the following form to derive the survival function *s* (Equation 7) related to soil water availability (*SWA*) depletion over time:

(7) $$\begin{array}{l} S_{SWA}=\frac{1}{1+e^{\left[{-ß}_{0}\left(SWA{-ß}_{1}\right)\right]}}, \end{array}$$

where two empirical parameters describe the growth rate (*ß*_0_) and the inflection point (*ß*_1_).

For the symmetric 2PL model used, the LD50_SWA_ values of the different provenances equate with inflection points (*ß*_1_) at *S*_SWA_ = 0.5 (Gregorczyk, 1991; SAS Institute Inc, 2014).

We tested the fitted models between the different populations for parallelism using a F-Test. The test compares the error sums-of-squares for a full and a reduced model. The full model gives each group different parameters. The reduced model forces the groups to share every parameter except for the inflection point. Moreover, the equality of model parameters across the levels of the populations, used as a grouping variable, was considered. With a comparison of parameter estimates (CPE), including an Analysis on Means (ANOM), the population means are tested against the overall mean.

The effect of decreasing soil water availability (SWA) on the plant internal water status, indicated by the predawn water potential, is indicative for the loss of water conductivity and cavitation, finally leading to hydraulic failure (e.g., Urli et al., 2013). Thus, besides relationships between LD50_SWA_ and climate variables at population origin also correlations between soil water availability (SWA) and mean predawn water potentials (Ψ_PD_) of the seedlings were analyzed by single linear regression analyses and F-test. Before the regression analysis (SWA vs. Ψ_PD_) we multiplied the Ψ_PD_ values by −1 to derive positive values and then log-transformed both parameters. A linear model was fitted, and values and model were then re-transformed [log (SWA, –Ψ_PD_)] resulting in a non-linear power function as a non-linear regression model. A bias correction was not applied. We tested the equality of the model across the populations using the already above mentioned tests on parallelism, CPE and ANOM. For all statistical analyses described and modeling purposes, *p* < 0.05 was considered significant.

## Results

### Soil water availability and seedling mortality

**Figure 2:**
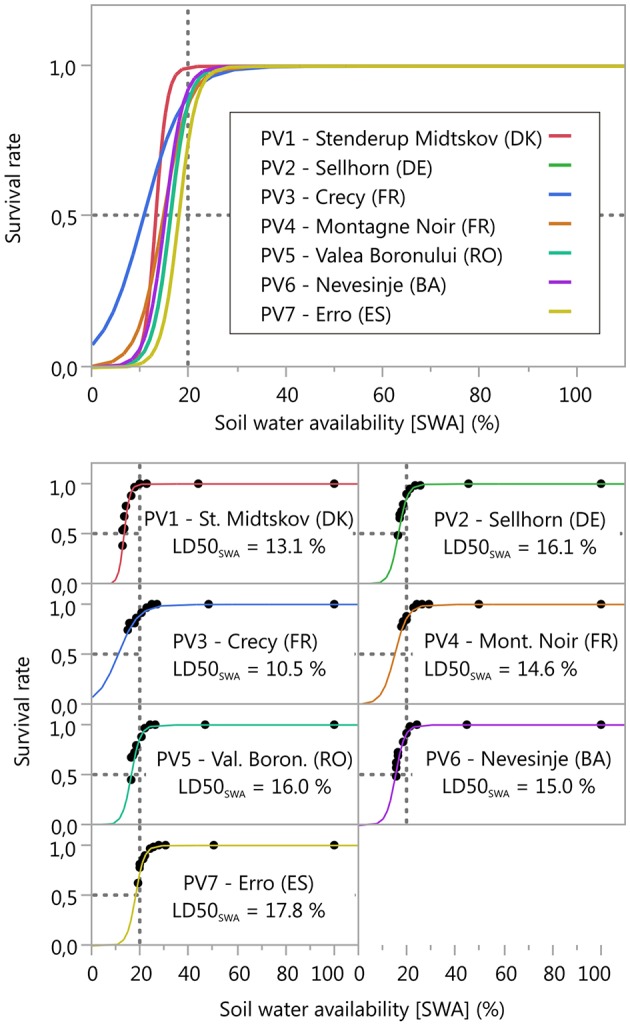
**Relationship between soil water availability [SWA] (%) and the survival rate, critical soil water availability (LDSOswA) derived from the 2PL model (50% mortality at the inflection point = LDSOswAI see chapter on analyses)**. The 20% SWA line found to be a critical threshold for plant performance by other studies (e.g., Granier et al., 2007; Domec et al., 2015) is displayed for orientation. Further information is shown in Table 3.

During the drought treatment, initial mortality of the seedlings was observed between 34 and 43 days from the commencement of the experiment. At the end of the experiment, mortality ranged between 33% (PV4, Montagne Noir, France) and 71% (PV1, Stenderup Midtskov, DK). A considerable increase in seedling mortality occurred when soil water availability (SWA) fell below values of 30–20% (Figure 2). However, differing responses between populations were found with respect to seedlings mortality dynamics under soil water depletion. The largest differences were found between PV1 (Stenderup Midtskov, DK) and PV3 (Crecy, FR). PV1 mortality started late (22% SWA), but had the strongest increase in mortality (growth rate ß_0_ ≈ 0.85, Table 3) overtaking all other populations in final mortality (0.62). In contrast, PV3 mortality began already at 27% SWA, followed by a retarded progress in mortality (growth rate ß_0_ ≈ 0.24, Table 3), not reaching 50% mortality at the end of the drought simulation. The mortality dynamics in terms of growth rate of the other populations were within this range. Correspondingly, growth rate (ß_0_) varies significantly from the overall mean parameter for PV1 (Stenderup Midtskov, DK) by exceeding the upper limit (UPL) and, for PV3 (Crecy, FR), by undershooting the lower limit (LWL) according to the comparison of parameter estimates (CPE, Table 3). The different shape of the fitted models (ß_0_) was also significant according to a parallelism F-test (F value 6.063, *p* < 0.0001).

**Table 3:** **Non-linear regression model parameters (growth rate ß_**0**_, inflection point ß_**1**_) and their standard error (SE) for predicting survival of beech seedlings from soil water availability (SWA), see Equation (4, 2PL) and Figure 2**.

	**PV1 Stenderup Midtskov**	**PV2 Sellhorn**	**PV3 Crecy**	**PV4 Montagne Noir**	**PV5 Valea Baronului**	**PV6 Nevesinje**	**PV7 Erro**
ß_0_ ± SE	**0.85 ± 0.10**	0.53 ± 0.06	**0.24 ± 0.05**	0.37 ± 0.08	0.54 ± 0.06	0.52 ± 0.06	0.56 ± 0.09
UPL	0.72	0.62	0.58	0.68	0.62	0.62	0.70
LWL	0.19	0.29	0.33	0.23	0.29	0.30	0.21
ß_1_ ± SE	**13.058 ± 0.0777**	**16.116 ± 0.183**	**10.525 ± 1.231**	14.592 ± 0.939	**16.030 ± 0.176**	**14.972 ± 0.160**	**17.797 ± 0.358**
UPL	14.347	14.630	17.428	16.647	14.610	14.567	15.097
LWL	13.932	13.650	10.852	11.632	13.671	13.713	13.183

*Parameter estimates are compared against the overall mean (CPE) with an Analysis of Means (ANOM), upper (UPL) and lower decision limit (LWL) is shown (α = 0.05). Bold parameter values (ß_*0*_, ß_*1*_) deviate significantly from the overall mean. Overall goodness of fit measures: Akaike information criterion AIC_C_ = −295.87, SSE = 0.059, MSE = 0.00093, r^*2*^ = 0.97*.

The LD50_SWA_ values corresponded to the inflection point of the model (ß_1_, Table 3). High LD50_SWA_ values were found for the populations PV2 (Sellhorn, DE), PV5 (Valea Boronului) and PV7 (Erro, ES), indicating high drought sensitivity (Figure 2). Low LD50_SWA_ values were found for PV3 (Crecy, FR) and also PV1 (Stenderup Midtskov, DK). LD50_SWA_ of all populations differed significantly from an overall mean except for PV4 (Montagne Noir, FR) looking on CPE results (Table 3).

The analyses revealed that seedlings' mortality dynamics and the critical threshold for drought impact indicated by LD50_SWA_ differ significantly among the selected populations. The most drought tolerant population in our experiment was PV3 (Crecy, FR) whereas the populations from higher elevations (PV5, PV6, and PV7) and northern origin (PV2) were drought sensitive. The most northern population (PV1, Stenderup Midtskov, DK) exhibited a remarkably strong drop in seedling survival that revealed sudden drought mortality risk for low SWA. A considerable extrapolation of 50% mortality is visible when applying the model to the two French populations (PV3, PV4), and thus the LD50_SWA_ values for both populations have to be considered with care. However, the extrapolated LD50_SWA_ values are supported by the clearly retarded mortality dynamics below 20% remaining SWA and the lower (negative) growth rate (ß_0_) of the regression model for both French populations compared to the other ones.

### Relationships between LD50_SWA_ and climate variables

**Figure 3:**
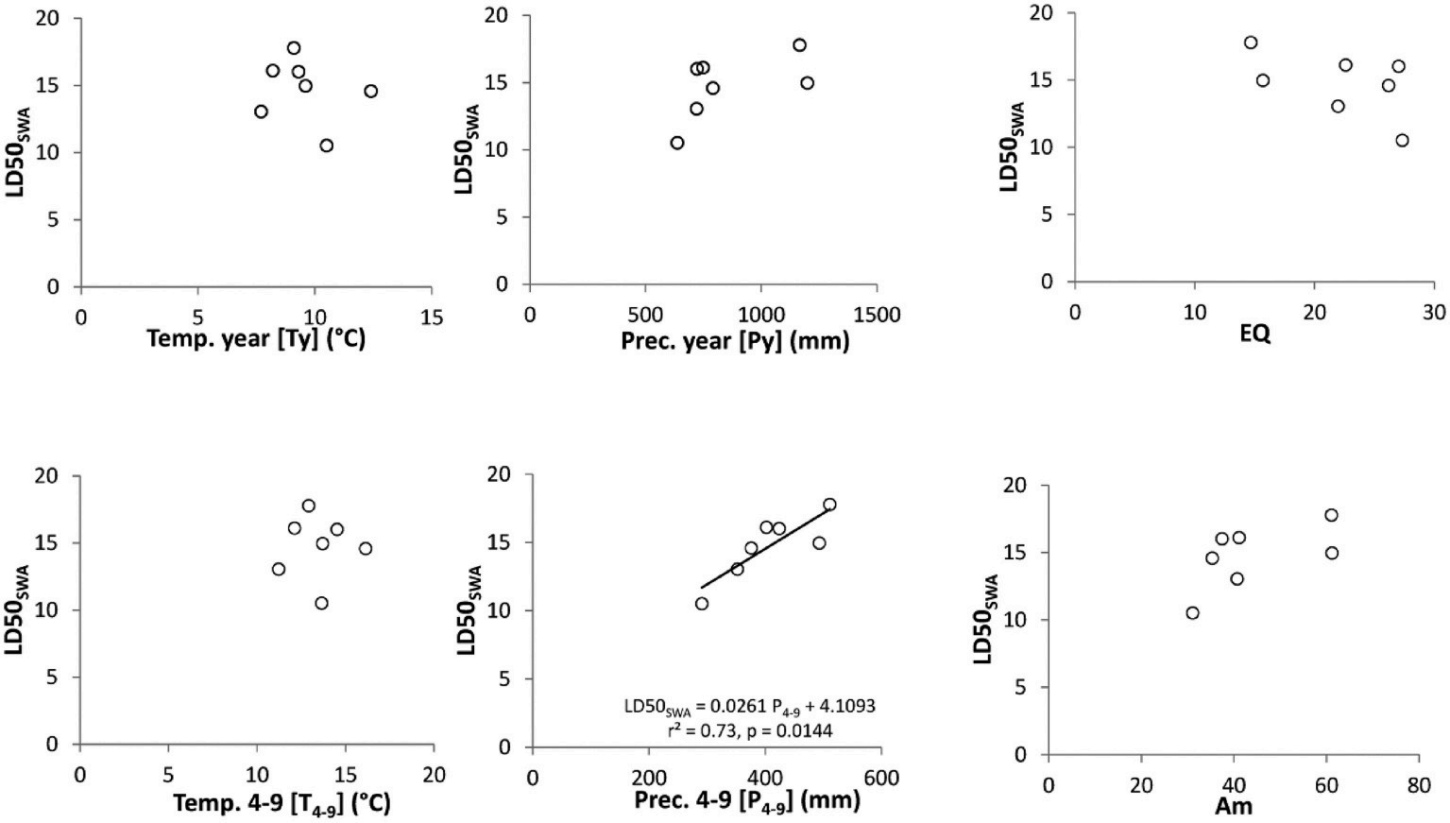
**Relationships between climate parameters and LD50_**SWA**_**. Left: temperature with mean annual temperature (Ty, above) and mean temperature during growing season (T_4−9_, below); middle: precipitation with mean annual precipitation (Py, above) and mean precipitation during growing season (P_4−9_, below); right: climate indices with Ellenberg Climate Quotient (EQ, above) and Aridity index of De Martonne (Am, below). The linear regression line displays a significant predictor effect of precipitation during the growing season (P_4−9_) on LDSOswA (seep values).

The critical soil water availability (LD50_SWA_) correlated significantly (*p* < 0.05, *r* = 0.73) with the mean growing season precipitation (Prec. 4–9, Figure 3, middle below). This relationship did not correspond to the geographical North-South gradient of the population origin, but is more influenced by the altitudinal precipitation gradient. No statistical relationships were found for temperature parameters (Ty, T_4−9_, T. max, latter not shown). Some tendencies are visible for mean annual precipitation sum (Py) and the climate indices used, which combined temperature and precipitation parameters (EQ, Am), but here the correlations between the climate parameter and the LD50_SWA_ values were not significant.

### Soil water availability and internal water status

**Figure 4:**
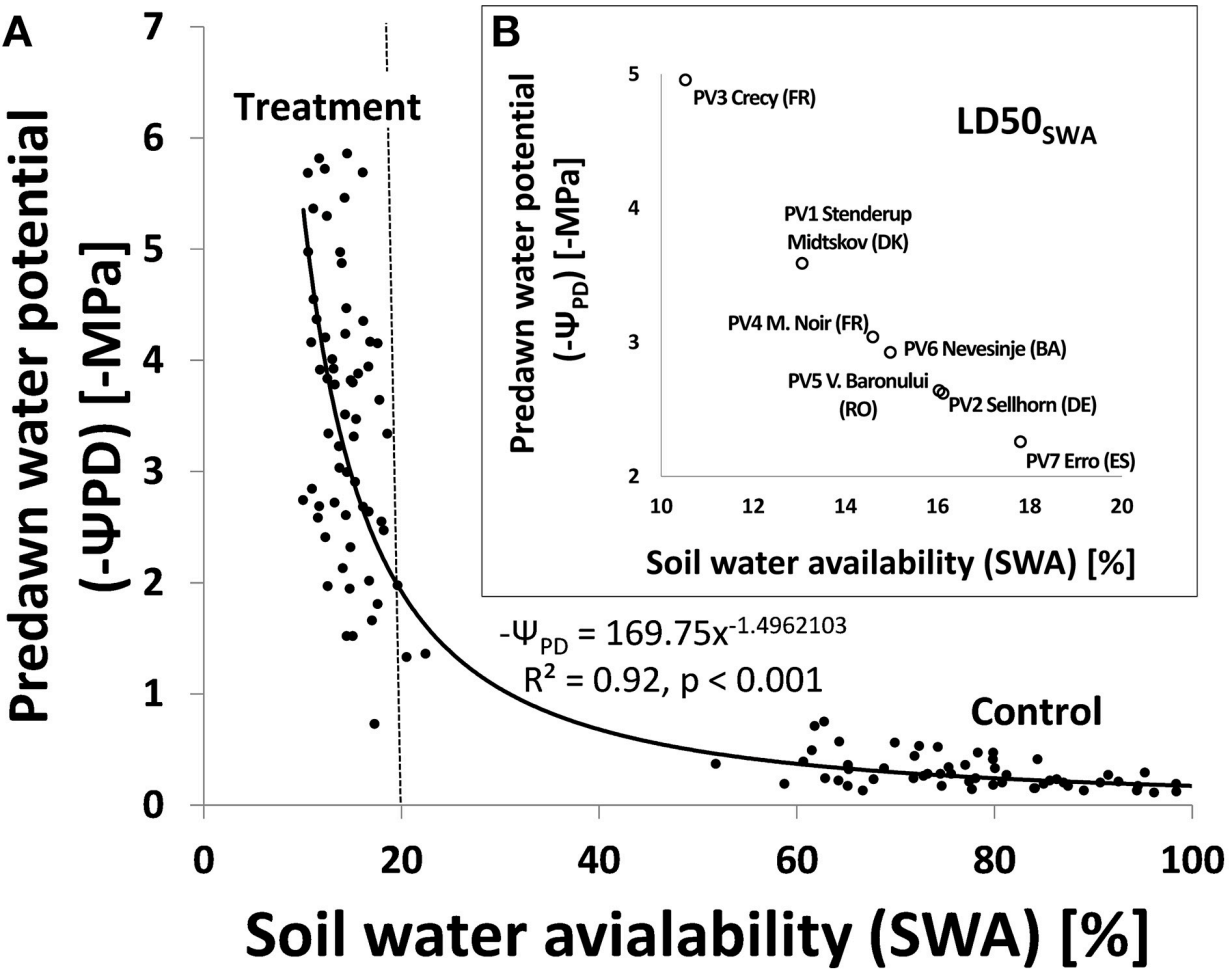
**(A)** Relationship between soil water availability (SWA) and negative predawn water potential (–ψ_PD_). Each point represents the population means at 9 dates from 33 to 61 days after the start of the drought experiment (treatment and control samples). Only seedlings with a predawn water potential > −6 MPa are included. The fitted model curve was derived from a linear regression analysis of Jog-transformed SWA and –ψ_PD_ values [log SWA = 2 1 2298002–1 1 4962103 log (–ψ_PD_)]. Values and model curve were then re-transformed [10 log^(swA, −ψPD)^] resulting in above displayed graph and power function; (B, inlaid figure) Estimated mean predawn water potentials when the different beech populations reached LDSO_SWA_ using the relationship described in Figure 4A.

The soil water availability (SWA) was closely correlated to the internal water status of the beech seedlings considered by the predawn water potentials (Ψ_PD_, Figure 4A). Due to observed heteroscedasticity the estimates are not unbiased. The figure shows population means of SWA and pre-dawn potentials of selected plants with signs of desiccation (treatment) or irrigated control plants (control) without drought stress. Plants without desiccation or indication of visible wilting during the drought treatment were not included. Mean values refer to nine dates during the experiment between the 33th and 61^st^ day after its start. A distinct change in Ψ_PD_values was visible when SWA dropped below 20%, corresponding to Ψ_PD_ of −2MPa. Small decreases in SWA below this threshold led to a strong drop in Ψ_PD_ values in wilting plants, which correspond to the mean mortality dynamics shown in Figure 2. In contrast to mean mortality dynamics, the tests on parallelism and equality of the model parameters gave no significant indication of variation across the populations (*p* < 0.05). Thus we used the general regression model to estimate the mean predawn water potentials (Ψ_PD_) when 50% mortality was reached (LD50_SWA_) for the different populations (Figure 4B, inlayed figure). The large variation of Ψ_PD_ values from nearly –5 MPa for PV3 (Crecy, FR) to –2.3 MPa for PV7 (Erro, ES) is induced by variation in LD50_SWA_ below 20% SWA.

## Discussion

### LD50 as a critical threshold of drought impact

The outcome of our study demonstrates that the derived LD50_SWA_ indicator is useful for analyzing the drought sensitivity of young trees. L50 was developed and first applied as a lethal dose or concentration indicator referring to 50% mortality of organism populations (Cavalli-Sforza, 1972) for dose-response analysis in the field of toxicology. In plant ecology, it was quite commonly used for lethal temperature (frost) impact on plants, including also trees (LT50, e.g., Zhang and Willison, 1987; Barranco et al., 2005; Kreyling et al., 2014; Hofmann et al., 2015). Some examples for the use of L50 approaches to indicate drought impact (LD50) considered exposure time only (Ivojević et al., 2012; Granda et al., 2015). Results of those studies, however, are only valid for the specific experimental environments used (e.g., pot size, soil substrate and plant material) and cannot be generalized or transferred to other environments. Kursar et al. (2009) presented an alternative approach of using leaf water status (relative leaf water content *RWC*, leaf water potentials Ψ) as a quantitative plant-related parameter for lethal drought assessment (LD50_RWC, Ψ_) providing more general results for tree species. Our LD50_SWA_ follows this idea in general, but uses soil water availability (SWA), which can be consistently assessed for different soil substrates in relation to different absolute available soil water amounts (cf. Meir et al., 2015). This provides new possibilities in soil water modeling for plant-related drought risk approaches (Bolte, 2015). However, a reference soil depth has to be defined describing the soil-root interface for water uptake, generally defined as effective rooting depth (ERD, cf. Czajkowski et al., 2009). This concept is supported by the simultaneous study of soil water and plant water status along the rooting gradient in mature oak stands in France, which reveals substantial water depletion dynamics down to the lower end of rooting zone corresponding to ERD (Bréda et al., 1995). In our experiment the entire pot depth that was completely rooted at the end of the experiment was regarded as ERD.

Our LD50_SWA_ indicator is a simplifying statistical indicator for drought impact at the population level, complementing and not replacing functional assessments and theories of extreme drought impact and plant mortality at an individual level (in particular hydraulic failure theory, Sperry et al., 1998; Brodribb and Cochard, 2009; Barigah et al., 2013). It also has to be considered in the context of other parameters like the mortality dynamics with decreasing SWA (Table 3, slope of regression model ß_*o*_). However, the L50_SWA_ result range of about 10–18% lethal soil water availability shown fits well to reported threshold of 20% available soil water told to induce strong effects, but not automatically mortality in mature trees and stands like the drop of whole tree hydraulic conductance (Domec et al., 2015) and the decrease in total ecosystem respiration *TER* (Granier et al., 2007). Thus, we regard LD50_SWA_ as a valid indicator that links plant-internal water status to soil hydraulics and by this provide novel possibilities for climate—soil water modeling and regionalisation of drought risk from plant to landscape and regional level. Recently, this approach was used for modeling the recent and future risk of lethal drought impact on beech regeneration by assessing period length below the LD50_SWA_ value under the canopy of mature stands of Norway spruce, Scots pine, and European beech on the national scale in Germany (Bolte, 2015).

### Different drought response of populations

Both our significant genetic differentiation of in LD50_SWA_ values (≈ 10–18%, Figure 2, Table 3) and the varying mortality dynamics (ß_0_, Table 3) among the different populations support the idea of local adaptation of populations within the European beech range. This is in line with many other studies on (1) leaf phenology (Wuehlisch et al., 1995; Chmura and Rozkowski, 2002; Nielsen and Jørgensen, 2003; Čufar et al., 2012; Robson et al., 2013), (2) cambium, xylem and phloem phenology (Prislan et al., 2013; Martínez del Castillo et al., 2016), (3) frost tolerance (Visnjic and Dohrenbusch, 2004; Czajkowski and Bolte, 2006b; Kreyling et al., 2014), and (4) drought response (Tognetti et al., 1995; García-Plazaola and Becerril, 2000; Peuke et al., 2002; Schraml and Rennenberg, 2002; Czajkowski and Bolte, 2006a; Rose et al., 2009; Ivojević et al., 2012; Eilmann et al., 2014; Thiel et al., 2014; Pšidová et al., 2015; Dounavi et al., 2016). Some studies, however, found indifferent or even contradicting results (Baudis et al., 2015; Hofmann et al., 2015) after comparing populations along a smaller geographic and climatic gradient within the continuous beech range (cf. Knutzen et al., 2015). Also Wortemann et al. (2011) found no evidence for genetic differentiation across beech populations for vulnerability to embolism by comparing European populations originating from the continuous distribution range of beech, only.

The adaptive potential of European beech, and other plant organisms, to drought and other climatic extreme events is triggered by two main processes: (1) genetic variation and/or (2) phenotypic plasticity (Meier and Leuschner, 2008; Lindner et al., 2010; Aranda et al., 2015). Genetic diversity of beech is mainly shaped by its phylogeographic history during the Pleistocene and Holocene (Harter et al., 2015). The isolated location of Pleistocene refuge areas and re-colonization pathways were indicative for large-scale genetic differentiation in Central European and Mediterranean distributions (Magri et al., 2006). Isolation during the highly variable interglacial climate conditions in the Pleistocene played a major role in increasing the genetic complexity of extant refuge populations, only partly preserved during the post-Pleistocene re-colonization toward north (“*southern complexity*” and “*northern purity*” paradigm, de Lafontaine et al., 2013). However, this interferes with recent evolutionary adaptation processes at the local level, occurring over only one or a few generations (Hamrick, 2004), when extreme weather events like droughts induce directed selection processes (Aitken et al., 2008; Spathelf et al., 2015). In particular for beech, marginal populations at the xeric distribution boundary are reputed to be the focus of local adaptation to drought, reducing genetic variation of local populations (Hampe and Petit, 2005) that exist in heterogeneous environments (Pluess et al., 2016).

The close correlation found between precipitation during the growing season (Prec. 4–9) at the population origins and the critical drought thresholds (LD50_SWA_) of the populations (Figure 3) suggests for local adaptation brought about mainly by recent evolutionary adaptation. This would explain also the fact that the actual precipitation conditions are indicative for the drought tolerance found and not the southern origin of the population near or even in Pleistocene refuge areas. In this sense, the distribution margin of beech, and thus the location of marginal beech populations, needs to be interpreted more in an ecological sense as beech occurrence near to its xeric limits rather than geographically by southern or eastern marginal location (cf. Hampe and Petit, 2005). This would mean that “ecologically” marginal populations due to local or regional xeric conditions may also occur within the continuous distribution range.

### Extreme drought adaptation, desiccation tolerance, and mortality of beech

Our findings underline the importance of assessing the adaptation of beech to drought at the intraspecific level. Hydraulic trait variations are seen as a major reason for different drought responses of tree populations within the species distribution range (Lamy et al., 2011; Balducci et al., 2015). Ecophysiological measurements (gas exchange, chlorophyll fluorescence) conducted alongside our drought experiment (Cocozza et al., personal communication) revealed differences in functional traits among the beech populations, but found no clear gradient in relation to location and climatic conditions at population origins. This addressed, however, mainly the drought response phase until complete stomata closure and considerable loss of hydraulic conductivity (at around 20% SWA and Ψ_PD_ ≈ −2 MPa, Figure 4A, cf. Hacke and Sauter, 1995; Cochard et al., 1999; Cruiziat et al., 2002). This is, however, decoupled from later desiccation and mortality dynamics (Delzon and Cochard, 2014). More than 90% loss of hydraulic conductivity of beech seedlings and young stands is reputed to be reached between −2.2 MPa (Magnani and Borghetti, 1995) and −4.0 MPa (Cochard et al., 1999). Advanced mortality in young beech was found at mean xylem water potentials of −4.5 MPa (Barigah et al., 2013, and this study). Furthermore, a recent study of mature beech in Germany revealed 88% of conductivity loss (P88) at xylem pressure means between −4.0 and −4.5 MPa (Schuldt et al., 2015). These findings fit well to our estimated variation of −2.3 MPa and nearly −5.0 MPa Ψ_PD_ at LD50_SWA_ when 50% mortality have occurred (Figure 4B). The outcome also strongly supports the idea of Delzon and Cochard (2014) that 50% mortality is linked to the almost complete loss of hydraulic conductivity (P88) in angiosperm trees like beech. Thus, LD50_SWA_ and P88 seem to represent corresponding indicators for lethal drought in beech and probably other angiosperms.

For plant survival under extreme drought, the ability to prolong the desiccation process and keep hydraulic integrity as long as possible seems to be a key adaptive issue (Bréda et al., 2006). In general, desiccation tolerance in plants involves the capacity to avoid deleterious effects of water shortage on the cellular membranes and maintain the bilayer structure in a xeric environment (e.g., Leprince et al., 1993). However, for taller vascular plants such as trees with complex hydraulic architecture, the resistance to cavitation and xylem embolism is by far the most important feature for desiccation tolerance (Lüttge et al., 2011). Our results suggest that there should be intra-specific variation in (1) morphological traits avoiding uncontrolled leaf water losses and/or (2) resistance to cavitation and hydraulic failure. Genetic variability in cavitation resistance is not clear for European beech, yet (Wortemann et al., 2011), but has been described in combination with morphological adaptation for Holm oak ecotypes (*Quercus ilex*, Peguero-Pina et al., 2014). Moreover, for beech populations significant differences in xylem anatomy (vessel size and vessel density) were found by Eilmann et al. (2014), which clearly point to higher drought resistance of a southern Bulgarian population from more xeric environments compared to those from mesic environments.

## Concluding remarks

Our study demonstrates that the introduced LD50_SWA_ indicator is a feasible indicator for critical soil water availability (SWA) in relation to plant desiccation and mortality. Thus, a residual SWA of 20% represents a critical limit (Granier et al., 2007), below which the risk of beech seedling mortality increases drastically. Also the correspondence of our LD50_SWA_ indicator with the P88 indicator found to describe a lethal water status in angiosperms (Delzon and Cochard, 2014) enables novel links for coupling ecophysiological and statistical mortality assessments. These insights provide new possibilities for local and regional modeling of drought risks based on soil water balance modeling. The significant intraspecific variation in survival under extreme drought (LD50_SWA_ and mortality dynamics) found can be used for the pre-selection of beech populations identified as especially apt for coping with the future climate. Further testing of these populations would be needed as well as more research on how this knowledge could apply in forest management aiming to increase our forests resistance to climate change. The differences revealed between “geographically” marginal and “climatically” marginal beech populations should be a matter of further research since common ideas of adaptive marginal populations may be biased, in particular due to the varying high-altitudinal location of southern population. Further research gaps include (1) the morphological and physiological background of genetic variation of adaptation and (2) the contribution of genetic variability and phenotypic plasticity to adaptive potentials of European beech.

## Author contributions

All authors (AB, TC, CC, RT, MM, EP, LubD, LucD, SD, HC, AR, ML, BC, CH and JM) contributed substantially to the writing of the manuscript. AB, TC, CC, RT, MM, EP, LubD, and JM drafted the conceptual design with the help of the author group and conducted the study. In addition, LucD, AR, ML, BC collected and delivered seed material for the study.

### Conflict of interest statement

The authors declare that the research was conducted in the absence of any commercial or financial relationships that could be construed as a potential conflict of interest.
